# Integrating miRNA, mRNA, and Targeted Metabolomics Analyses to Explore the Regulatory Mechanism of Cardiac Remodeling in Yili Horses

**DOI:** 10.3390/biology14111535

**Published:** 2025-11-01

**Authors:** Tongliang Wang, Xixi Yang, Wanlu Ren, Jun Meng, Xinkui Yao, Hongzhong Chu, Runchen Yao, Manjun Zhai, Yaqi Zeng

**Affiliations:** 1 College of Animal Science, Xinjiang Agricultural University, Urumqi 830052, China; wtl13639911402@163.com (T.W.); xxyang2022@126.com (X.Y.); renwanlu@xjau.edu.cn (W.R.); junm86@xjau.edu.cn (J.M.); yaoxinkui@xjau.edu.cn (X.Y.); zhaimanjun@yeah.net (M.Z.); 2 Xinjiang Key Laboratory of Horse Breeding and Exercise Physiology, Urumqi 830052, China; 3 Horse Industry Research Institute, Xinjiang Agricultural University, Urumqi 830052, China; 4 Xinjiang Yili Kazakh Autonomous Prefecture Animal Husbandry Station, Urumqi 835000, China; 13364712998@163.com (H.C.); m18095936088@163.com (R.Y.)

**Keywords:** cardiac remodeling, multi-omics analysis, RNA-seq, Yili horses

## Abstract

**Simple Summary:**

Training improves not only the athletic performance of horses but also promotes beneficial changes in heart structure and function. This study examined how different states of training affect the hearts of Yili horses, a breed known for its endurance and strength. We compared agility group, ordinary group, and untrained group using heart imaging, genetic analysis, and metabolic profiling. Our results show that training leads to adaptive heart remodeling, enhances energy metabolism, and activates specific signaling pathways that support heart health. Key regulatory molecules were identified that help explain how exercise benefits the equine heart. These findings provide valuable insights for optimizing training programs and improving cardiac care in racing horses.

**Abstract:**

Training not only enhances the athletic performance of horses but also improves cardiac structure and function, strengthens cardiovascular adaptability, and reduces the risk of cardiovascular diseases. However, the consequences of training on equine cardiac structure and function remain unclear. This study investigated the morphological, functional, genetic, and metabolic changes in the hearts of Yili horses divided into three groups: high athletic performance (agility group, AG), low athletic performance (ordinary group, OG), and untrained (untrained group, UN). The results showed remodeling of the cardiac structure and physiological adaptations in AG and OG compared to UN groups, with differences between AG and OG primarily in the left ventricle. To explore the molecular mechanisms underlying these phenotypic changes, transcriptomic and metabolomic analyses (particularly GO and KEGG pathway analyses) were performed to assess differences in gene expression and metabolite levels among the three groups. Our results show that miR-1842, miR-671, miR-106b and miR-18a were differentially expressed in the trained groups (AG group and OG group) compared with the control group that did not receive training. These regulatory factors would regulate PFKFB3 to affect the glycolytic activity mediated by HIF-1, there by promoting glycolysis and changing lactate level. This, in turn, would positively feedback to stabilize HIF-1, thus forming a closed loop for the reprogramming of myocardial energy metabolism. In the AG group, positive effects of cAMP signaling were noticeable. In conclusion, our findings offer new insights into physiological cardiac remodeling in Yili horses by highlighting genetic and metabolomic changes resulting from exercise training.

## 1. Introduction

Yili horse is a prominent breed in western China well-known for its endurance, explosive power, and adaptability, making it a popular choice for racing and high-intensity riding activities. In recent years, with the growing popularity of Chinese equestrian sports in China and internationally, the demand for Yili horses has significantly increased. Due to its unique physiological traits and superior athletic performance, studying its cardiac adaptation mechanisms during training is of significant scientific and practical importance [1,2,3]. Investigating the cardiac remodeling process in Yili horses during this phase not only provides a scientific basis for improving their training outcomes but also offers valuable insights for optimizing training strategies in other sport horse breeds.

Cardiac remodeling is a common physiological adaptation that occurs during the training process of sport horses, such as racehorses or jumping horses. It involves structural and functional adjustments of the heart to cope with prolonged or intense physical training [4]. This adaptive cardiac remodeling enhances the pumping efficiency and athletic performance of horses. However, sustained high-intensity cardiac loads may lead to potential pathological changes. Therefore, understanding the molecular mechanisms underlying cardiac remodeling is crucial for optimizing training programs for race horses, reducing cardiac strain, and improving health management.

In the regulation of cardiac remodeling, miRNAs are key non-coding RNA molecules (approximately 20–24 nucleotides) that regulate gene expression by binding to the 3′ untranslated region (3′UTR) of target mRNAs [5]. miRNAs play critical roles in physiological processes such as cardiomyocyte proliferation, apoptosis, fibrosis, and energy metabolism. They are involved in the regulation of pathological myocardial hypertrophy and heart failure and play a significant role in adaptive cardiac remodeling induced by exercise [6,7]. Therefore, it is essential to investigate the molecular regulatory roles of miRNAs in adaptive cardiac remodeling. Metabolism not only supports the normal function of cardiomyocytes but also modulates cardiac adaptive remodeling through metabolic intermediates. Exercise training significantly impacts cardiac metabolism, including fatty acid synthesis, oxidation, phospholipids, triglycerides, cholesterol, glycolysis, branched-chain amino acid degradation [8], mitochondrial homeostasis, and the pentose phosphate pathway [9]. Lipid metabolism imbalance has been identified as a driving factor in pathological cardiac changes [9]. Consequently, understanding the role of lipid metabolism in cardiac remodeling has practical implications for preventing exercise-induced cardiac injuries.

Increasing evidence indicates that miRNA-mRNA interactions play a pivotal role in regulating cardiac adaptive remodeling. Specifically, miRNAs can regulate the activity of lipid metabolism pathways by targeting lipid metabolism-related mRNAs, thereby maintaining energy metabolism balance in the heart under exercise conditions [10,11]. Additionally, changes in lipid metabolism may feedback to regulate miRNA expression, forming a complex interaction network [12]. This synergistic interplay among miRNAs, mRNAs, and lipid metabolism underpins the molecular basis of cardiac adaptive remodeling. Unveiling these mechanisms offers novel perspectives on the process of exercise-induced cardiac remodeling.

## 2. Materials and Methods

### 2.1. Ethics Statement

The protocol of this study was reviewed and approved by the Animal Policy and Welfare Committee of Xinjiang Agricultural University (Approval No. 2023037). All experimental procedures strictly adhered to relevant animal welfare and ethical guidelines. Informed consent was obtained from the horse owners.

### 2.2. Experimental Design and Horse Grouping

**Figure 1:**
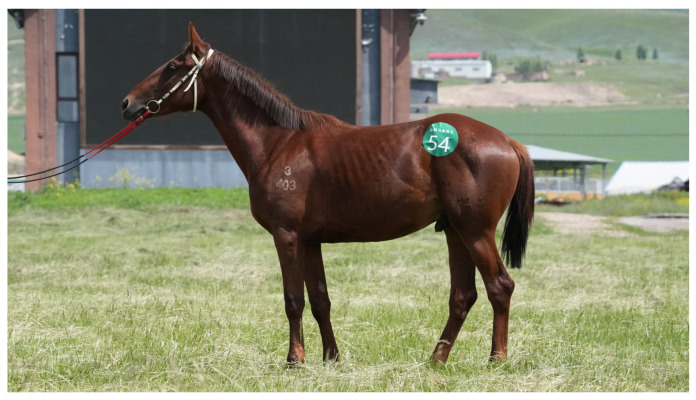
Presentation of the experimental horses.

A total of 26 Yili horses, aged 18 months, were selected for this study (Figure 1) These horses were sourced from the national-owned stud farm in Yili, Xinjiang Uygur Autonomous Region and were chosen based on close birth dates, similar body structure, and same feeding and management. All the horses were trained properly. Subsequently, they underwent a six-month exercise training program based on the following structure. The exercise intensity was monitored using HR, with a target maximum HR (HRmax) set at 240 bpm, Trot at 50–60% HRmax, canter at 60–70% HRmax, and gallop at 70–80% HRmax. Detailed information about the training program is provided in Supplementary Text S1.

At the end of the training period, the performance of the horses was tested by three 1000 m races. Based on the results, the top ten performers were assigned to the AG (average time: 75.11 ± 1.44 s), and the lower ten performers were assigned to the OG (average time: 83.94 ± 2.21 s). The race result of 1000 m were highly significant difference between AG and OG (Supplementary Table S1).

### 2.3. Echocardiographic Assessment

Echocardiographic data were collected for all horses in a resting state using a color Doppler ultrasound system (Mindray M6, Shenzhen, China) to evaluate cardiac structure and functional parameters. Prior to imaging, the right side of the sternum was cleaned, and imaging was conducted using a 2.5 MHz transducer. The maximum imaging depth was set at 30 cm, with a maximum angle of 110°. Three non-consecutive imaging were taken by a professional staff to capture images of the heart at end-diastole and end-systole. The imaging modes included B-mode right parasternal long axis, B-mode right parasternal left ventricular outflow tract, and B/M-mode right parasternal short axis, capturing both static and dynamic images. Heart rates during imaging ranged from 32 to 45 bpm. Parameters measured included left ventricular internal diameter at end-diastole (LVIDd), left ventricular internal diameter at end-systole (LVIDs), and ejection fraction (EF). To ensure reliability, the measurements were cross-referenced with existing literature and adjusted for the specific cardiac characteristics of Yili horses. For instance, the average LVIDd values were 5.8 cm in the AG, 5.6 cm in the OG, and 5.3 cm in the UN group. Data analysis focused on parameters closely related to cardiac remodeling with complete datasets retained for evaluation.

### 2.4. Transcriptomic Analysis

#### 2.4.1. RNA Extraction

Blood samples (5 mL) were collected from the jugular vein of each horse using EDTA anticoagulant tubes while the horses were in a resting state. and RNA was extracted using Trizol reagent for subsequent experiments. The samples were aliquoted into cryovials and immediately stored in liquid nitrogen for subsequent analysis. Then in the laboratory, The RNA extraction method is provided in the Supplementary Text S2.

#### 2.4.2. mRNA and miRNA Library Construction and Sequencing

Extracted RNA samples were used to construct mRNA and miRNA libraries. The mRNA libraries were prepared by enriching mRNA with poly(A) tails, while the miRNA libraries were built by selecting small RNA fragments 18–30 nt in length. The constructed libraries were initially quantified using the Qubit dye-based method and assessed for insert size using the Qsep400 Fragment Analyzer(BiOptic, Inc., New Taipei City, Taiwan) to confirm the expected range of 200–300 bp. Qualified libraries were subjected to high-throughput sequencing on the Illumina HiSeq™ 2500 platform, generating single-end (SE) or paired-end (PE) reads of 50 bp or 150 bp.

#### 2.4.3. mRNA Identification

Raw sequencing data were processed to remove low-quality reads, adapter-contaminated reads, and reads with high proportions of unknown bases (N), resulting in clean data. Clean reads were aligned to the reference genome (GCF_002863925.1_EquCab3.0_genomic.fna) using HISAT2 2.2.1, achieving a mapping rate above 70% to ensure reliability. Reads were assembled into transcripts using StringTie software 2.2.1, and gene expression levels were quantified using the FPKM (Fragments Per Kilobase of transcript per Million mapped reads) method. Differential expression analysis was performed using the DESeq2 software 1.38.3, with screening criteria of |log_2_ Fold Change| ≥ 1 and *p*-value < 0.05.

#### 2.4.4. Functional Annotation and Pathway Analysis of DEmRNAs

To interpret the biological significance of differentially expressed genes (DEGs), GOseq was used for Gene Ontology (GO) enrichment analysis, identifying significantly enriched GO terms in Biological Processes (BP), Molecular Functions (MF), and Cellular Components (CC). KEGG pathway analysis was conducted using the KOBAS tool, with a threshold of *p* < 0.05, to identify pathways significantly enriched in metabolism and signal transduction.

#### 2.4.5. miRNA Identification

High-quality clean data were processed to remove 3′ adapters and low-quality sequences, and small RNA fragments (18–26 nt) were selected. miRNA identification was performed using ACGT101-miR (v4.2) software. The identified miRNA sequences were compared to the miRBase database to confirm miRNA identities.

#### 2.4.6. miRNA Target Gene Prediction

Target genes of differentially expressed miRNAs (DEmiRNAs) were predicted using TargetScan 7.2 and miRanda software 3.3a. Prediction criteria included a TargetScan context score percentile ≥ 50 and miRanda maximum free energy (Max Energy) < −10 kcal/mol. The final target gene set was obtained from the intersection of results from the two methods.

#### 2.4.7. Correlation Analysis of DEmRNAs and DEmiRNAs

Differentially expressed miRNAs were paired with their target mRNAs to analyze negative regulatory relationships. Pearson correlation analysis was used to identify significantly negatively correlated miRNA-mRNA pairs. Regulatory networks were visualized using the iGraph package in R to illustrate the molecular interaction networks.

#### 2.4.8. RT-qPCR Validation of mRNAs and miRNAs

The transcriptome data of this study originated from the long-term exercise study on the effects of exercise on the cardiac structure and function, blood transcriptome, and plasma metabolome of horses, which was conducted in 2023. The experimental design, treatment conditions, and basic background of this batch of samples have been elaborated in detail in previous studies. All samples have been processed according to the experimental protocols after sequencing and preliminary qPCR verification, and no remaining samples are retained. The original sequencing files of this batch of data have been uploaded to the NCBI SRA database (accession number: [SUB 15457665]). The detailed sample preparation, sequencing process, and qPCR verification methods have been published in “A multi-omics-based study on the mechanism of exercise-induced cardiac remodeling and anti-fibrosis in Yili horses” [13].

### 2.5. Metabolomics Analysis

#### 2.5.1. Data Collection and Processing

Blood samples (5 mL) were collected from the jugular vein of each horse in a resting state. The samples were centrifuged at 3000× *g* for 10 min to obtain plasma, which was then aliquoted into cryovials and stored at −80 °C until further analysis.

#### 2.5.2. Targeted Metabolomics Analysis

Metabolomics analysis was conducted using an ultra-high-performance liquid chromatography-tandem mass spectrometry (UPLC-MS/MS) system. After thawing, samples were vortexed for 10 s to mix thoroughly. A 50 μL aliquot of each sample was transferred to a labeled centrifuge tube, followed by the addition of 300 μL of 20% acetonitrile-methanol extraction solution containing internal standards. The samples were vortexed for 3 min, centrifuged at 15,000× *g* for 10 min at 4 °C, and 200 μL of the supernatant was transferred to another labeled tube. After standing at −20 °C for 30 min, the samples were centrifuged again at 15,000× *g* for 3 min at 4 °C, and 180 μL of the supernatant was transferred to a vial insert for analysis. Further details about T3 chromatographic conditions are provided in Supplementary Text S3.

### 2.6. Data Analysis

Differential metabolites were screened using both univariate and multivariate methods, including hypothesis testing, fold change (FC) analysis, and orthogonal partial least squares discriminant analysis (OPLS-DA). Screening criteria were set as VIP > 1, *p* < 0.05, and FC ≥ 2 or ≤0.5. Metabolites significantly associated with the training status were identified. Metabolites were annotated using the Kyoto Encyclopedia of Genes and Genomes (KEGG) pathway database. Further details are provided in Supplementary Text S4.

### 2.7. Statistical Analysis

All graphs were generated using GraphPad Prism 8.0 (GraphPad Software Inc., San Diego, CA, USA). Statistical analysis of the data was performed using SPSS 26.0 (IBM, Armonk, NY, USA). Data were expressed as mean ± standard deviation. Differences between groups were analyzed using one-way ANOVA. Homogeneity of variance within groups was tested, with *p* > 0.05 indicating no significant difference.

## 3. Results

### 3.1. Cardiac Function Assessment of Yili Horses—Characteristics of Cardiac Remodeling

The echocardiographic measurements taken in the resting state are summarized in Table 1. These results highlight differences among the three groups in parameters such as left ventricular internal diameter at end-diastole (LVIDd), left ventricular internal diameter at end-systole (LVIDs), and ejection fraction (EF). Detailed cardiac structural parameters for each group are provided in Supplementary Table S2.

### 3.2. miRNA and mRNA Expression Analysis

#### 3.2.1. Overview of Blood Sequencing Data from Yili Horses

Gene expression of the 26 samples was analyzed using high-throughput sequencing technology. A total of 1,452,462,002 raw reads were obtained, with 1,268,623,014 clean reads retained after filtering. The average error rate for the samples was below 0.01%, and the Q20 and Q30 values were over 98% and 94%, respectively, indicating high data quality. Additionally, the GC content ranged from 49.93% to 51.73%, showing stable quality across all sample groups. Sequence alignment with the reference genome revealed an average alignment efficiency of 94.5%, with an independent alignment rate of 90.9%, further ensuring the accuracy and usability of the data.

#### 3.2.2. Differential mRNA Analysis

**Figure 2:**
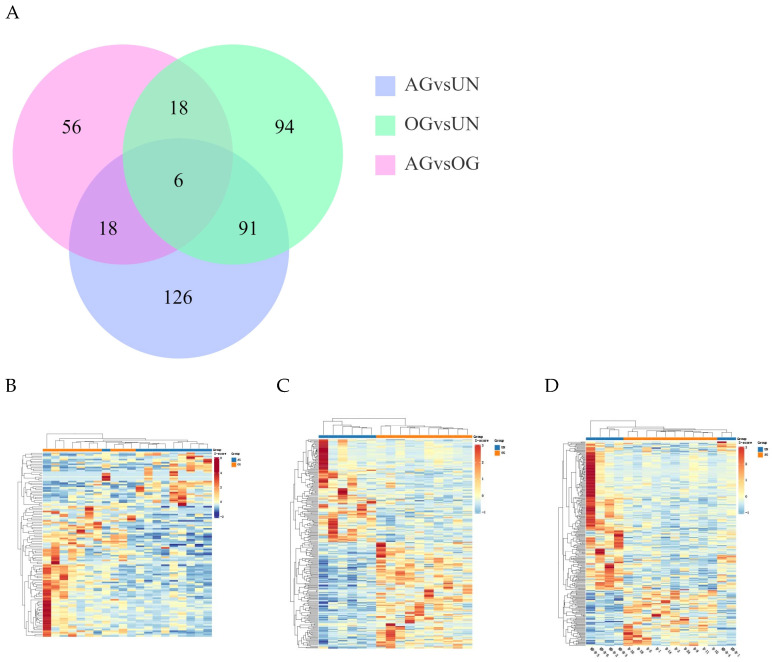
Analysis of DEGs in blood samples among the three groups of Yili horses. (**A**) The Venn diagram shows the total number of differentially expressed genes (DEGs) in the AG, OG and UN groups. (**B**–**D**) These three groups’ heat map clustering diagrams of differentially expressed genes.

The clean reads were aligned to the reference genome (https://ftp.ncbi.nlm.nih.gov/genomes/all/GCF/002/863/925/GCF_002863925.1_EquCab3.0/, accessed on 1 March 2024) using HISAT2 software 2.2.1. A total of 593 differentially expressed genes (DEGs) were identified in the blood samples among the three groups (Figure 2A). Compared to the AG, 98 DEGs were identified in the OG, and 241 DEGs were identified in the UN group. Between the OG and UN groups, 209 DEGs were identified. As shown in the figure, the differences between the AG and UN groups were greater than those between the OG and UN groups, indicating that training had a notable impact on gene expression. The differences between the AG and OG were smaller, suggesting that athletic performance had a common effect on gene expression. Hierarchical clustering analysis was performed on the DEGs to visually display the changes in gene expression. The clustering heatmaps for the differentially expressed genes between the groups are shown in Figure 2B–D. The DEGs among the three groups are provided in Supplementary Table S3.

#### 3.2.3. Differential miRNA Analysis

**Figure 3:**
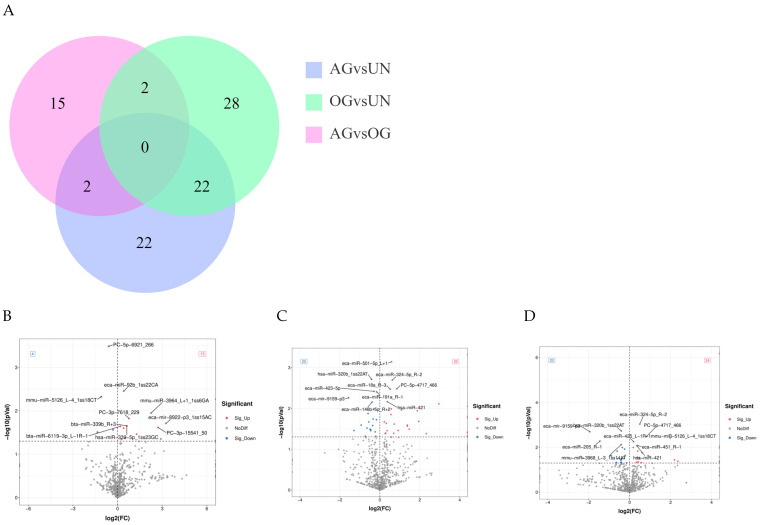
Differential miRNA analysis (**A**) Analysis of DEmiRNAs among the three groups of Yili horses. (**B**–**D**) Volcano plots of the distribution of significantly upregulated and downregulated miRNAs in the comparisons between the groups.

To identify known miRNAs in the blood of Yili horses, miRNA identification was performed using ACGT101-miR based on miRBase and the species genome. A total of 117 differentially expressed miRNAs (DEmiRNAs) were identified. Among these, 19 DEmiRNAs were found between the OG and AG. Between the OG and UN groups, 52 DEmiRNAs were identified. In the AG vs. UN group comparison, 46 DEmiRNAs were identified (Figure 3A). Volcano plot analysis further revealed the expression patterns of these miRNAs across groups, suggesting their potential roles in regulating the exercise process (Figure 3B–D). Similarly, in comparing the three groups, the common differences between the trained group and the untrained group will receive more attention. The differential miRNAs among the three groups are provided in Supplementary Table S4.

Target gene prediction for miRNAs was conducted based on their mechanisms of action in animals and plants. Differentially expressed miRNAs were analyzed using TargetScan (v5.0) [14,15,16] and miRanda (v3.3a) [17,18,19], with the final predicted target genes obtained as the intersection of results from the two tools.

### 3.3. Differential Expression Analysis

#### 3.3.1. GO Functional Annotation and KEGG Enrichment Analysis of DEGs

To investigate the effects of conditioning and training on cardiac gene expression in Yili horses, comprehensive Gene Ontology (GO) functional annotation and KEGG pathway enrichment analyses were performed for the DEGs, thereby to explore the biological processes and signal transduction pathways influenced by training in Yili horses.

#### 3.3.2. GO and KEGG Functional Annotation Analysis

**Figure 4:**
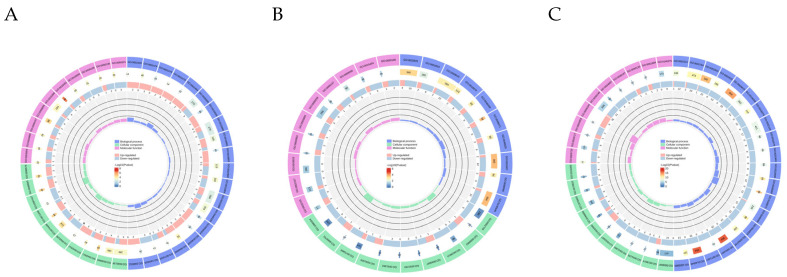

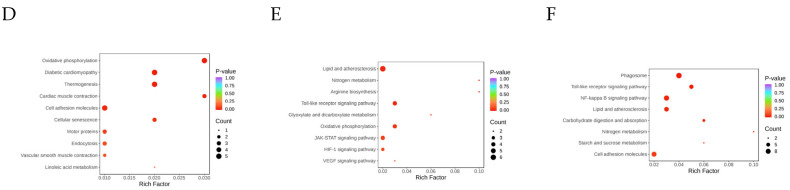
Functional annotation analysis of DEGs in the blood of Yili horses across the three groups. (**A**–**C**) Top 40 significantly enriched GO functional annotations of differentially expressed genes (DEGs) in the AG vs. OG, OG vs. UN, and AG vs. UN comparisons, respectively. (**D**–**F**) Top 10 key KEGG pathway enrichment analyses of DEGs in the AG vs. OG, OG vs. UN, and AG vs. UN comparisons, respectively.

GO analysis of the DEGs revealed an enrichment of the terms “cell membranes and organelles” (cellular component), “metabolism”, biological regulation” and “intracellular processes” (Biological process) and “binding”, “catalysis” and “molecular transport” (Molecular Function) (Figure 4A–C). Specifically, the genes were primarily enriched in pathways related to redox reactions and electron transport, antigen processing and presentation in MHC complexes, and viral defense and immune responses. KEGG pathway analysis showed that DEGs among the three groups were mainly enriched in pathways related to Environmental Information Processing, Organismal Systems, Cellular Processes, and Metabolism. For the OG vs. AG, DEGs were significantly enriched in pathways such as Oxidative Phosphorylation, Cardiac Muscle Contraction, and Viral Myocarditis, which provide the energetic and material basis for myocardial cell function. For the OG vs. UN and AG vs. UN group comparisons, DEGs were significantly enriched in pathways including Lipid and Atherosclerosis, Nitrogen Metabolism, and the NF-kappa B Signaling Pathway. These pathways are closely related to vascular relaxation and the maintenance of cardiac function.

#### 3.3.3. DEmRNA-DEmiRNA Correlation Analysis and Functional Analysis of Target Genes

**Figure 5:**
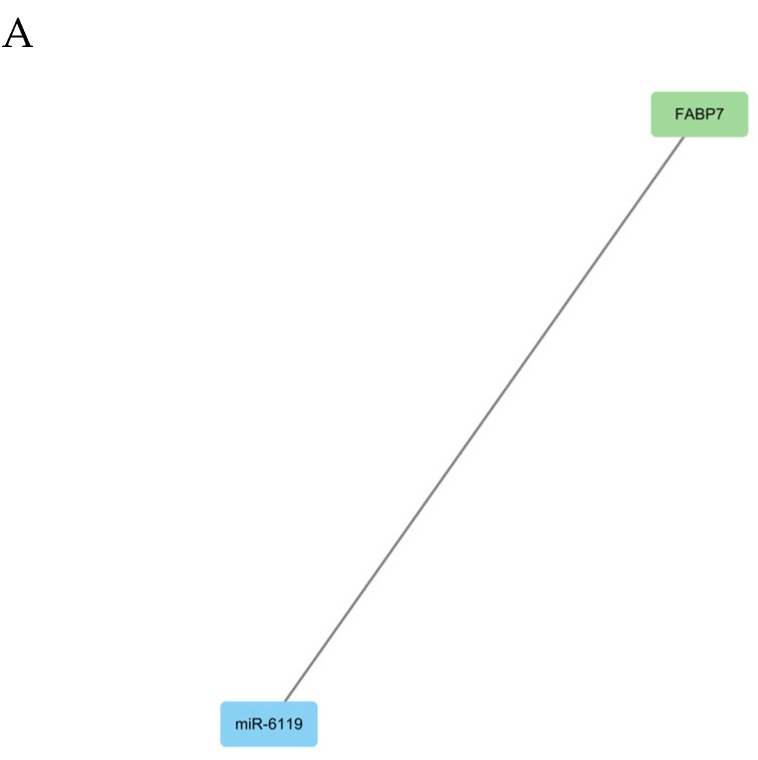

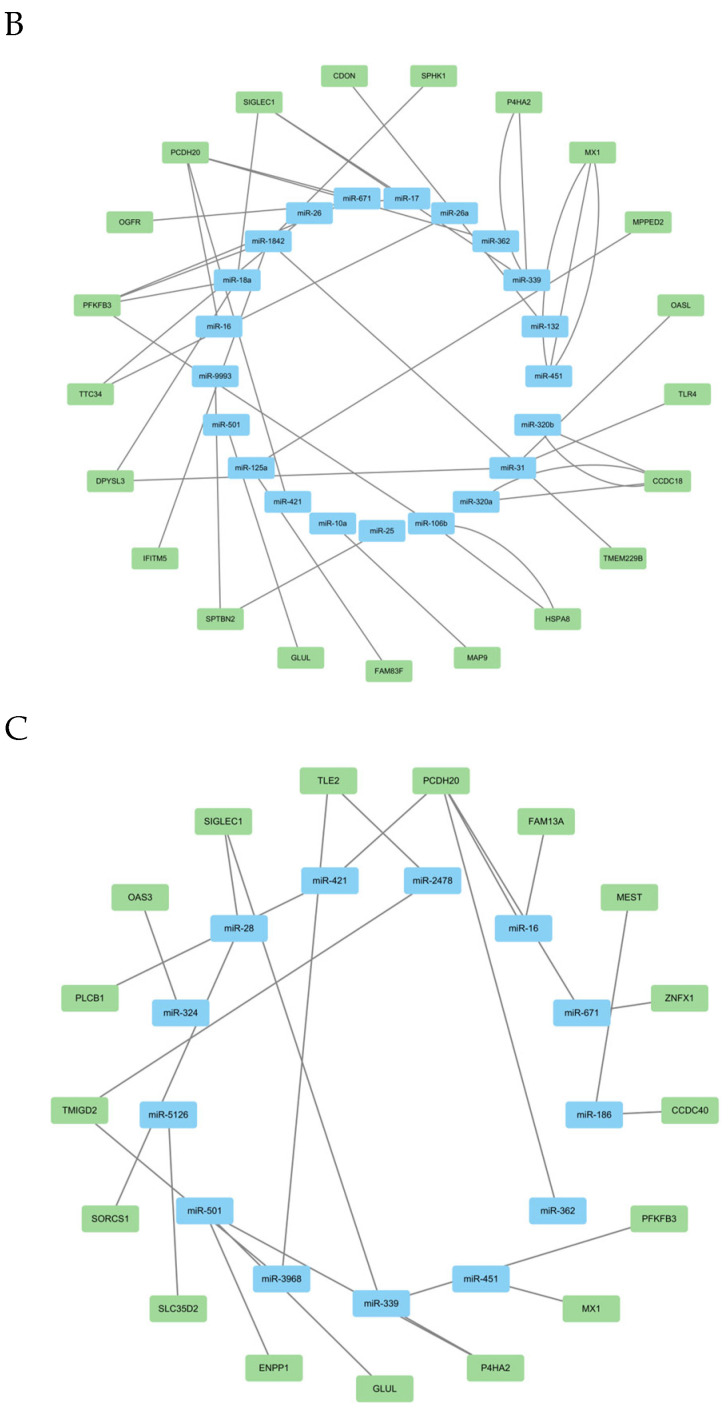
Co-expression network of DEmiRNAs and DEmRNAs in the blood of Yili horses among the three groups. (**A**) AG vs. OG comparison. (**B**) OG vs. UN comparison. (**C**) AG vs. UN comparison.

To explore the role of mRNAs and miRNAs in physiological cardiac remodeling, the target genes of miRNAs were cross-referenced with the mRNA transcriptome obtained via RNAseq. Negatively co-expressed mRNA-miRNA pairs were identified. In the OG vs. AG comparison, one miRNA was upregulated, corresponding to one downregulated mRNA. In the OG vs. UN comparison, 48 mRNA-miRNA relationship pairs were identified, while 38 pairs were identified in the AG vs. UN comparison. The co-expression network revealed that miR-1842, miR-671, miR-106b, and miR-18a collectively regulate PFKFB3. miR-362, miR-671, miR-421 and miR-16 jointly regulate PCDH20 (Figure 5A–C).

### 3.4. Broad Targeted Metabolomics

**Figure 6:**
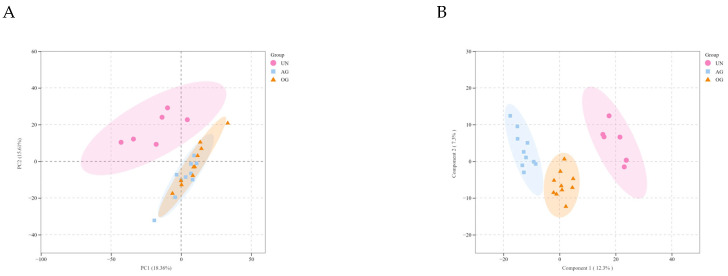

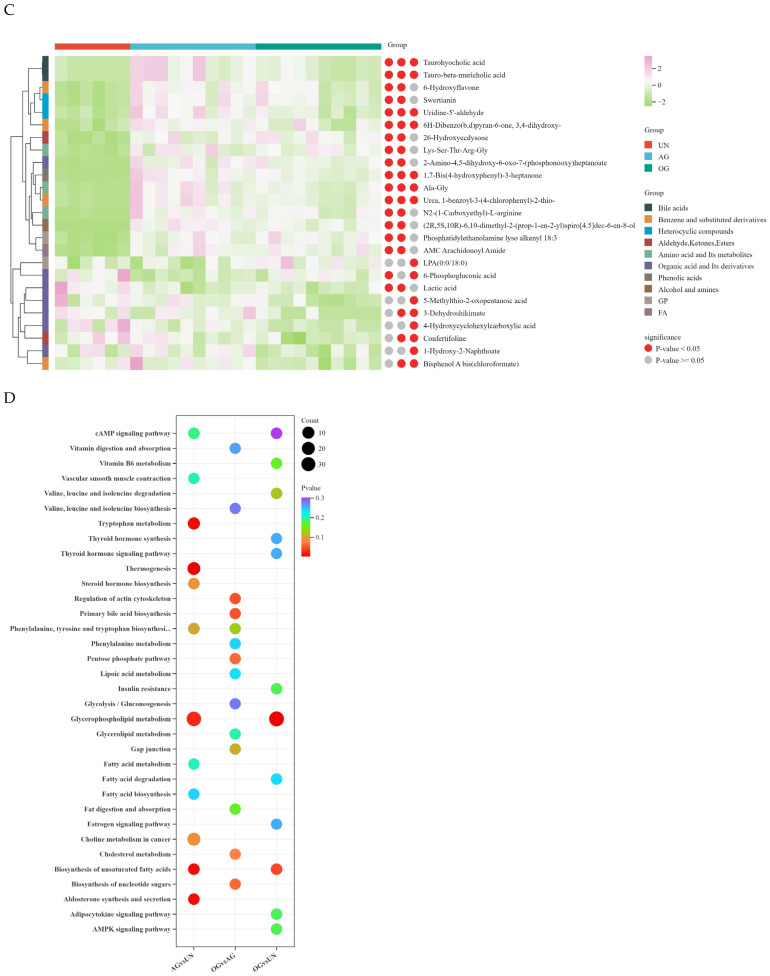
Plasma metabolomics analysis among the three groups of Yili horses. (**A**) PCA analysis of AG vs. OG, OG vs. UN, and AG vs. UN comparisons. (**B**) OPLS-DA score plots of AG vs. OG, OG vs. UN, and AG vs. UN comparisons. (**C**) PCA analysis of AG vs. OG, OG vs. UN, and AG vs. UN comparisons. (**D**) KEGG enrichment analysis of the top 10 key pathways for AG vs. OG, OG vs. UN, and AG vs. UN comparisons.

PCA revealed minimal variability between the OG and AG, suggesting that horses with different athletic performance share certain common metabolic characteristics. However, significant differences were observed between the other groups (Figure 6A). OPLS-DA and S-plots were used to establish score plots for the groups (Figure 6B). Based on the OPLS-DA model, metabolites with a Variable Importance in Projection (VIP) score > 1 and *p* < 0.05 were identified as significantly different.

The results showed that, between the OG and AG, 69 differential metabolites were identified, with 6 upregulated and 63 downregulated; between the OG and UN groups, 317 differential metabolites were identified, with 105 upregulated and 212 downregulated; between the AG and UN groups, 346 differential metabolites were identified, with 175 upregulated and 171 downregulated. The results indicate that the number of differential metabolites increases with improved athletic performance (Figure 6C).

We identified differential metabolites with high VIP values, including organic acids and their derivatives, glycerophospholipids, fatty acyls, as well as amino acids and their metabolites, which were found to be highly abundant. These metabolites are likely closely associated with horse training. We focused on the differences between the trained and the untrained groups, and paid special attention to changes in metabolic processes during exercise, such as lactic acid accumulation.

KEGG enrichment analysis revealed that differential metabolites between the OG and AG were primarily associated with pathways such as the regulation of actin cytoskeleton, biosynthesis of nucleotide sugars, and the pentose phosphate pathway (Figure 6D), which play crucial roles in muscle contraction, cardiac development, and functional maintenance. Between the OG and UN groups, differential metabolites were enriched in pathways including glycerophospholipid metabolism, valine, leucine, isoleucine degradation, and the AMPK signaling pathway, all of which are important for regulating heart function and exercise performance. Meanwhile, between the AG and UN groups, differential metabolites were enriched in pathways related to the biosynthesis of unsaturated fatty acids, phenylalanine, tyrosine, tryptophan biosynthesis, and the cAMP signaling pathway, all of which are essential for maintaining normal physiological functions and influencing cardiac electrophysiology. The differential metabolites among the three groups are provided in Supplementary Table S5.

### 3.5. Integrated Transcriptomic and Metabolomic Analysis

**Figure 7:**
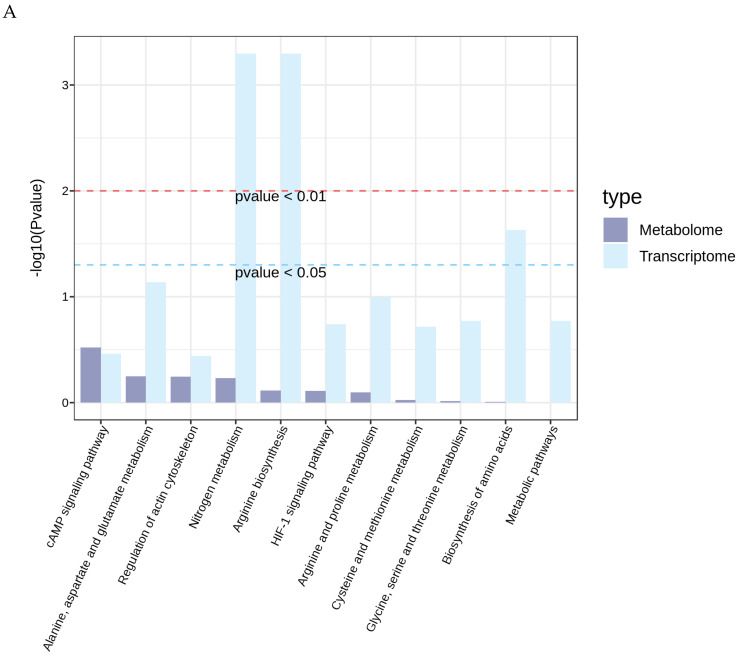

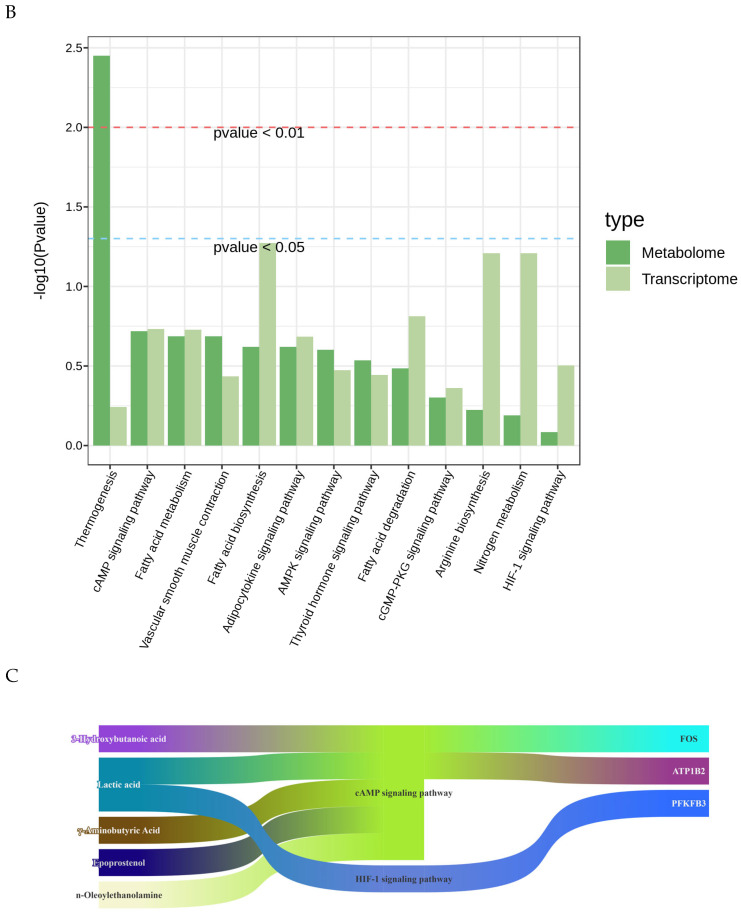
KEGG pathway analysis integrating transcriptomic and metabolomic data. (**A**) OG vs. UN comparison. (**B**) AG vs. UN comparison. Sankey diagram of KEGG pathway-gene-metabolites interactions. (**C**) AG vs. UN comparison.

We mapped all DEGs and DEMs obtained from the transcriptomic and metabolomic analyses onto the KEGG pathway database to identify their shared pathways. This approach allowed us to determine the major biochemical and signal transduction pathways jointly involved by DEGs and DEMs. No shared enriched pathways were identified between the OG and AG. However, comparison between the OG and UN groups revealed shared enriched pathways such as the HIF-1 signaling pathway, cAMP signaling pathway, and Metabolic pathways. Similarly, comparison between the AG and UN groups identified shared enriched pathways including the AMPK signaling pathway, Thyroid hormone signaling pathway, cGMP-PKG signaling pathway, and Metabolic pathways. The interactions between the genes and metabolites in these pathways play significant roles in cardiac remodeling (Figure 7A,B). Furthermore, we will present the key pathways that are common to both the trained and untrained groups, in order to effectively observe the synergistic effects of mRNA and metabolites within the pathways (Figure 7C). As shown in the figure, 3-Hydroxybutanoic acid, Lactic acid, Aminobutyric Acid, Epoprostenol, n-Oleoylethanolamine, along with FOS, ATP1B2, and PFKFB3, participate in the regulation of the heart through the cAMP signaling pathway and the HIF-1 signaling pathway.

## 4. Discussion

This study revealed that conditioning and training significantly promote adaptive changes in horse cardiac structure and function through incremental training of Yili horses. Such structural remodeling leads to adaptive alterations in heart size, shape, and function, consistent with previous studies that reported positive cardiac remodeling induced by exercise [20]. Moreover, this study elucidated the specific molecular adaptive mechanisms in high-performance horses. Training not only optimizes cardiac energy metabolism and membrane stability but also improves cardiac health by regulating inflammatory responses and myocardial fibrosis. These findings provided new insights into the molecular mechanisms of exercise training.

By comparing Yili horses from the untrained group (UN), ordinary group (OG), and agility group (AG) over nine months of incremental training, our study found that the left ventricular internal diameter at end-diastole (LVIDd) and end-systole (LVIDs) in the trained groups (AG and OG) were significantly larger than those in the untrained group (UN). Furthermore, the LVIDd and LVIDs values in the AG were slightly higher than those in the OG, indicating a positive correlation between left ventricular diameter expansion and athletic performance. Previous studies have shown that exercise training enhances the efficiency of cardiac output distribution, making horses one of the animals with the highest peak oxygen consumption rates [21]. Another study reported that the heart mass of horses accounts for approximately 0.9–1% of their body weight, a proportion higher than that of many other species, and that left ventricular size and systolic function are significantly correlated with athletic performance [22]. In this study, we also observed an increase in interventricular septum thickness (IVS) in the trained groups, which contributes to enhanced left ventricular contractility and pumping efficiency, consistent with prior research on humans [23]. Additionally, our findings indicated that prolonged high-intensity exercise led to a significant increase in right ventricular internal diameter at end-diastole (RVDd), although no significant differences were observed from the data analysis. Meanwhlie, the right ventricular internal diameter at end-systole (RVDs) remained unchanged. Further analysis showed that the aortic diameter (AOD) and left ventricular fractional shortening (FS%) in the AG were significantly better than those in the OG. These results suggest that ventricular remodeling and blood flow distribution are more prominent in high-performance horses. Long-term endurance training may significantly improve myocardial elasticity and strength by enhancing myocardial contractility, consistent with other studies showing increased myocardial contractility following endurance training [24].

This study also observed characteristics of the “athlete’s heart” in the trained group, characterized by significantly reduced heart rates and adaptive remodeling of ventricular structure and function. The trained groups exhibited significantly better values in parameters such as end-diastolic volume (EDV), end-systolic volume (ESV), stroke volume (SV), and left ventricular mass (LVM) compared to the untrained group, with the agility group (AG) showing even more pronounced optimization of cardiac structure and function. These findings indicate that training significantly improves cardiac adaptive remodeling and athletic performance in horses.

To further explore the molecular mechanisms underlying cardiac remodeling in Yili horses induced by exercise training, this study analyzed blood transcriptome data from the trained and untrained groups. The results showed that the MYH14 gene was significantly upregulated in the group with a higher degree of cardiac remodeling (log_2_ FC = 3.35). Previous studies have reported that MYH14, a member of the myosin family, is closely related to muscle development and function. It also regulates the composition of the extracellular matrix, inhibits myocardial fibrosis, and protects the heart [25]. Another gene, SPHK1, was significantly expressed in the trained groups (log_2_ FC = −3.4). SPHK1 catalyzes the conversion of sphingosine to sphingosine-1-phosphate (S1P), which activates the PI3K/Akt signaling pathway through S1P receptors, promoting cardiac angiogenesis and repair. Additionally, SPHK1 has been identified as a key regulatory factor in endothelial cells [26]. In the comparison between the AG and UN groups. Combined analysis of miRNA and mRNA revealed miR-1842, miR-671, miR-106b, and miR-18a. and miR-339 collectively regulate PFKFB3, Enzymes associated with the PFKFB3 gene play a pivotal role in cellular energy homeostasis. Mechanistic studies indicate that the TGF-β1 signaling pathway may trigger the release of intracellular PFKFB3 protein from myofibroblasts via a caspase-dependent apoptotic pathway by regulating the programmed cell death process in cardiac fibroblasts [27]. Functional experiments confirm that exogenous recombinant PFKFB3 protein not only significantly drives cardiac fibroblast differentiation toward a myofibroblast phenotype but also enhances chemotactic migration of M0 macrophages via the PI3K/Akt signaling axis. This protein exerts a pronounced regulatory effect on macrophage polarization states, simultaneously stimulating both M0 and M1 subsets to secrete proinflammatory factors such as IL-6 and TNF-α, suggesting its multifaceted regulatory role within the cardiac fibrosis microenvironment [28]. Interestingly, miR-671, miR-362, miR-16, and miR-421 were found to regulate PCDH20 in both AG vs. UN and OG vs. UN. The PCDH20 gene belongs to the non-clustering cadherin subfamily within the cadherin family [29]. PCDH20 likely causes abnormal zebrafish heart development by promoting apoptosis. HIF-1 is a key regulator of ECM remodeling under hypoxic conditions. It directly influences ECM composition, organization, and mechanical properties by activating gene expression of collagen hydroxylases (P4HA1, P4HA2) and lysyl hydroxylases (PLOD2). Findings in this study suggest that eca-miR-421 may participate in regulating cardiac function by targeting PCDH20 [30]. These findings suggest that training not only strengthens myocardial structure but also optimizes cardiac adaptability by regulating angiogenesis and cellular repair. The trained groups (AG and OG) also shared the significant expression of FOS, TLR4, and IRF7. Research suggests that high expression of FOS may initiate the expression of myocardial structural protein genes, acting as a trigger for myocardial hypertrophy [31]. TLR4 is involved in regulating myocardial fibrosis and hypertrophy by modulating pro-inflammatory factors such as interleukin-6 (IL-6) [32]. Meanwhile, IRF7 mitigates pathological cardiac hypertrophy and fibrosis caused by pressure overload by inhibiting the NF-κB signaling pathway [33]. These results indicate that the coordinated expression of these genes in the trained groups demonstrates that exercise training not only promotes physiological adaptation of the myocardium but also finely regulates remodeling processes to avoid excessive remodeling. This further optimizes the structural and functional adaptability of the heart, providing essential support for high-intensity exercise.

Through transcriptomic GO and KEGG analyses, broad-targeted metabolomics KEGG analysis, and integrated transcriptomic-metabolomic analysis, this study further demonstrated the regulatory effects of exercise training on cardiac metabolism and signaling pathways. Compared to the untrained group, the trained groups exhibited significant enrichment in energy metabolism-related pathways such as oxidative phosphorylation and glycerophospholipid metabolism, which are primarily involved in energy supply for cardiomyocytes and stabilization of cell membranes. Additionally, significant enrichment in lipid metabolism pathways, including lipid and atherosclerosis and linoleic acid metabolism, highlighted the critical role of training in optimizing myocardial energy metabolism and balancing fatty acid metabolism. The agility group (AG) displayed a unique molecular regulatory pattern in cardiac metabolic adaptation. Pathways significantly enriched in the AG included cardiac muscle contraction, adrenergic signaling in cardiomyocytes, and the MAPK signaling pathway, reflecting the group’s adaptation to higher-intensity exercise by enhancing cardiac pumping capacity and optimizing myocardial excitability regulation [34]. Furthermore, significant changes in the calcium signaling pathway and the PI3K-Akt signaling pathway revealed the AG’s advantages in suppressing myocardial fibrosis and regulating metabolism. These findings align with previous studies [35], suggesting that exercise training can significantly promote energy metabolism, structural adaptation, and functional optimization of the heart by regulating metabolic and signaling pathways.

The focus is on the comprehensive analysis of the multi-omics data of the AG and UN groups., it was discovered that PFKFB3-lactic acid-HIF-1α positive feedback loop is the core mechanism of cellular metabolic reprogramming and plays a crucial role in responding to hypoxic stress and tissue repair. The excellent performance group of this loop exhibits significant specificity: including precise regulation of PFKFB3, enhanced utilization efficiency of lactic acid, and increased stability efficiency of HIF-1α. These characteristics enable the excellent performance group to rapidly establish adaptive metabolic reprogramming, efficiently maintain energy homeostasis and tissue repair. This provides new theoretical support for understanding the mechanisms of cardiac adaptation to exercise training.

## 5. Conclusions

In summary, this study revealed the significant effects of long-term incremental training on the cardiac structure, function, and molecular mechanisms of Yili horses. Training markedly promoted myocardial hypertrophy and proliferation, and myocardial angiogenesis, resulting in positive adaptive cardiac remodeling. The agility group (AG) exhibited more significant advantages in cardiac remodeling and metabolic regulation, including enhanced myocardial contractility, improved energy metabolism efficiency, and optimized lipid metabolism. These findings suggest that specific signaling pathways, such as cAMP signaling pathway and HIF-1 signaling pathway, play critical roles in precise regulation of cardiac metabolic adaptability and structural–functional optimization. The co-expression network analysis identifies that miR-1842, miR-671, miR-106b, and miR-18a synergistically regulate PFKFB3, and the PFKFB3-lactic acid-HIF-1α positive feedback loop serves as the core mechanism of cellular metabolic reprogramming, which plays a critical role in mediating hypoxic stress response and tissue repair.

## Figures and Tables

**Table 1:** Results of echocardiography of the three performance groups of Yili horses under resting conditions showing cardiac structural and functional measurements.

TERM	Untrained Group (UN)	Ordinary Group (OG)	Agility Group (AG)
RVDd	2.38 ± 0.13 ^a^	2.6 ± 0.22 ^a^	2.6 ± 0.25 ^a^
IVSd	2.14 ± 0.22 ^b^	2.41 ± 0.22 ^a^	2.51 ± 0.31 ^a^
LVIDd	8.77 ± 0.22 ^c^	9.56 ± 0.27 ^b^	10.04 ± 0.19 ^a^
LVFWd	1.79 ± 0.26 ^b^	1.97 ± 0.22 ^a^	2.03 ± 0.17 ^a^
RVDs	1.66 ± 0.13 ^a^	1.72 ± 0.24 ^a^	1.76 ± 0.26 ^a^
IVSs	3.52 ± 0.25 ^b^	3.92 ± 0.29 ^a^	4.06 ± 0.31 ^a^
LVIDS	5.35 ± 0.11 ^b^	5.71 ± 0.17 ^a^	5.86 ± 0.13 ^a^
LVFWs	2.64 ± 0.26 ^b^	3 ± 0.34 ^a^	3.09 ± 0.31 ^a^
LADd	8.17 ± 0.49 ^b^	8.63 ± 0.46 ^a^	9.07 ± 0.57 ^a^
LADs	9.32 ± 0.52 ^b^	10.23 ± 0.49 ^a^	10.36 ± 0.54 ^a^
AODd	4.62 ± 0.24 ^c^	5.11 ± 0.14 ^b^	5.35 ± 0.18 ^a^
PADd	3.89 ± 0.23 ^b^	4.2 ± 0.24 ^a^	4.22 ± 0.12 ^a^
PADs	4.29 ± 0.22 ^b^	4.75 ± 0.36 ^a^	4.83 ± 0.23 ^a^
EF	0.61 ± 0.06 ^b^	0.65 ± 0.07 ^ab^	0.68 ± 0.06 ^a^
FS	0.39 ± 0.01 ^c^	0.4 ± 0.01 ^b^	0.42 ± 0.02 ^a^
LVminor	14 ± 0.57 ^b^	14.92 ± 0.41 ^a^	14.77 ± 0.43 ^a^
SV	285.07 ± 17.98 ^c^	350.93 ± 22.67 ^b^	398.9 ± 24.78 ^a^
EDV	423.35 ± 23.81 ^c^	511.47 ± 31.39 ^b^	569.21 ± 23.9 ^a^
ESV	138.28 ± 6.42 ^b^	160.54 ± 10.9 ^a^	170.31 ± 8.8 ^a^
CO	12,830.48 ± 1030.97 ^b^	18,248.57 ± 2172.95 ^a^	18,309.83 ± 1867.84 ^a^
LVM	1422.91 ± 246.18 ^c^	1895.89 ± 211.19 ^b^	2160.79 ± 225.55 ^a^
HR	55.23 ± 2.73 ^a^	46.97 ± 2.66 ^b^	45.93 ± 4.28 ^b^

Note: UN, untrained Control group, *n* = 6; OG, low performers group, *n* = 10; AG, high performers group, *n* = 10. RVDd: End-diastolic right ventricular diameter; IVSd: End-diastolic interventricular septal thickness; LVIDd: End-diastolic left ventricular diameter; LVFWd: End-diastolic left ventricular free wall thickness; LADd: End-diastolic left atrial diameter; LADs: End-systolic left atrial diameter; AODd: End-diastolic aortic root diameter; PADd: End-diastolic pulmonary artery diameter; PADs: End-systolic pulmonary artery diameter; EF: Ejection fraction; FS: Fractional shortening; LVminor: Left ventricular minor; SV: Stroke volume; EDV: End-diastolic left ventricular volume; ESV: End-systolic left ventricular volume; CO: Cardiac output; LVM: Left ventricular myocardial mass; HR: Heart rate. Statistical comparisons of echocardiography results from the three performance groups of Yili horses. If the letters labeling the columns are different, it means that a certain substance is significantly different in the two groups. If the letters are the same, it means that there is no significant difference, and the letters themselves have no special meaning.

## Data Availability

The original contributions presented in the study are included in the article/Supplementary Material, further inquiries can be directed to the corresponding author.

## Supplementary Materials

The following supporting information can be downloaded at: https://www.mdpi.com/article/10.3390/biology14111535/s1, Text S1: Training Plan; Text S2: Lipid LC-MSMS Analysis Experimental Method; Text S3: analysis method; Text S4: Data analysis; Table S1: Yili Horse 1000m Speed Race Results; Table S2: Yili Horse Cardiac structure and function data; Table S3: Differential gene list among the three groups; Table S4: Differential miRNA list among the three groups; Table S5: Differential metabolites list among the three groups.
