# Supplementary material for: Integrating miRNA, mRNA, and Targeted Metabolomics Analyses to Explore the Regulatory Mechanism of Cardiac Remodeling in Yili Horses
Source: Biology (Basel). 2025 Nov 1;14(11):1535. doi: 10.3390/biology14111535 (PMC12650387; doi:10.3390/biology14111535)
Supplement: Supplementary file 1 [file biology-14-01535-s001.zip › Supplement Text 2 Lipid LC-MSMS Analysis Experimental Method.pdf]

Supplement text 2

1、Chemicals and reagent

HPLC-grade acetonitrile(ACN), methanol (MeOH), isopropanol (IPA), dichloromethane (CH2Cl2), tert-butyl methyl ether (MTBE)werepurchased from Merck (Darmstadt, Germany).HPLC-grade formic acid (FA), Ammonium formate (AmFA) were purchased from Sigma-Aldrich (St. Louis, MO. USA). Ultrapure water was obtained bya Milli-Q system (Millipore, Billerica, MA). Lipid standardswere purchased from Sigma-Aldrich orAvanti Polar Lipids (Alabaster,AL).

2、Sample preparation and extraction

The sample was taken out from the -80℃ refrigerator, thawed on ice and vortexed for 10 s. Mix 50 µL of the sample and 1mL of the extraction solvent (MTBE: MeOH =3:1, v/v) containing internal standard mixture. After whirling the mixture for 15 min, 200 µL of ultrapure water was added. Vortex for 1 min and centrifuge at 12,000 rpm for 10 min. 200 µL of the upper organic layer was collected and evaporated using a vacuum concentrator. The dry extract was dissolved in 200 µL reconstituted solution (ACN: IPA=1:1, v/v) to LC-MS/MS analysis.

3、HPLC Conditions

The sample extracts were analyzed using an LC-ESI-MS/MS system (UPLC, ExionLC AD， https://sciex.com.cn/ ; MS, QTRAP® 6500+ System, https://sciex.com/). The analytical conditions were as follows, UPLC: column, Thermo Accucore™C30 (2.6 µm, 2.1 mm×100 mm i.d.); solvent system, A: acetonitrile/water (60/40,V/V, 0.1% formic acid, 10 mmol/L ammonium formate), B: acetonitrile/isopropanol (10/90 VV/V, 0.1% formic acid, 10 mmol/L ammonium formate); gradient program, A/B（80:20, V/V）at 0 min, 70:30 V/V at 2.0 min, 40:60 V/V at 4 min, 15:85 V/V at 9 min, 10:90 V/V at 14 min, 5:95 V/V at 15.5 min, 5:95 V/V at 17.3 min, 80:20 V/V at 17.3 min, 80:20 V/V at 20 min; flow rate, 0.35 ml/min; temperature, 45℃; injection volume: 2 µL. The effluent was alternatively connected to an ESI-triple quadrupole-linear ion trap (QTRAP)-MS.

4、ESI-MS/MS Conditions

LIT and triple quadrupole (QQQ) scans were acquired on a triple quadrupole-linear ion trap mass spectrometer (QTRAP), QTRAP® 6500+ LC-MS/MS System, equipped with an ESI Turbo Ion-Spray interface, operating in positive and negative ion mode and controlled by Analyst 1.6.3 software (Sciex). The ESI source operation parameters were as follows: ion source, turbo spray; source temperature 500 ℃; ion spray voltage (IS) 5500 V（Positive）,-4500 V(Neagtive); Ion source gas 1 (GS1), gas 2 (GS2), curtain gas (CUR) were set at 45, 55, and 35 psi, respectively. Instrument tuning and mass calibration were performed with 10 and 100 µmol/L polypropylene glycol solutions in QQQ and LIT modes, respectively. QQQ scans were acquired as MRM experiments with collision gas (nitrogen) set to 5 psi. DP and CE for individual MRM transitions was done with further DP and CE optimization. A specific set of MRM transitions were monitored for each period according to the metabolites eluted within this period.
