# Supplementary material for: Integrating miRNA, mRNA, and Targeted Metabolomics Analyses to Explore the Regulatory Mechanism of Cardiac Remodeling in Yili Horses
Source: Biology (Basel). 2025 Nov 1;14(11):1535. doi: 10.3390/biology14111535 (PMC12650387; doi:10.3390/biology14111535)
Supplement: Supplementary file 1 [file biology-14-01535-s001.zip › Supplementary Text 1 Training Plan.pdf]

Training Plan

Week 1: Desensitization training (both groups participated initially; however, only the training group continued after this week)

Education phase:

1. Teach horse to listen with lead exercises;
2. Practice exercises to make horse comfortable with touch;
3. Train your horse to follow directions through circle work;
4. Introducing your horse to a saddle;
5. Place the saddle on your horse's back;
6. Tighten the girth in intervals;
7. Use a mounting block to climb in the saddle;
8. Mount and dismount from the saddle in 10-min intervals.

Begin walking your horse while in the saddle.

Week 2: thirty minutes in the lunge ring (walk—5 min, trot—20 min, and walk—5 min);

Week 3: forty minutes in the lunge ring (walk—5 min, trot—30 min, and walk—5 min);

Week 4: one hour on the horse walker (2 m/s—20 min, 3 m/s—30 min, and 4 m/s—10 min);

Training phase, starting from Week 5:

Week 5: thirty-five minutes of warm-up on the horse walker (2 m/s—5 min, 3 m/s—15 min, and 4 m/s—10 min), five minutes of riding in the training arena (trot—5 min);

Week 6: thirty-five minutes of warm-up on the horse walker (2 m/s—5 min, 3 m/s—15 min, and 4 m/s—10 min), ten minutes of riding in the training arena (trot—10 min);

Week 7: thirty-five minutes of warm-up on the horse walker (2 m/s—5 min, 3 m/s—15 min, and 4 m/s—10 min), fifteen minutes of riding in the training arena (trot—15 min);

Week 8: thirty-five minutes of warm-up on the horse walker (2 m/s—5 min, 3 m/s—

15 min, and 4 m/s—10 min), fifteen minutes of riding in the training arena (trot—20 min);

Week 9: thirty-five minutes of warm-up on the horse walker (2 m/s—5 min, 3 m/s—15 min, and 4 m/s—15 min), trot—5 min and canter— 10 min, gallop—0 min

Week 10: thirty-five minutes of warm-up on the horse walker (2 m/s—5 min, 3 m/s—15 min, and 4 m/s—15 min), trot—10 min and canter— 10 min, gallop—0 min

Week 11: thirty-five minutes of warm-up on the horse walker (2 m/s—5 min, 3 m/s—15 min, and 4 m/s—15 min), trot—15 min and canter— 10 min, gallop—0 min

Week 12: thirty-five minutes of warm-up on the horse walker (2 m/s—5 min, 3 m/s—15 min, and 4 m/s—15 min), trot—20 min and canter— 10 min, gallop—0 min

Week 13: thirty-five minutes of warm-up on the horse walker (2 m/s—5 min, 3 m/s—15 min, and 4 m/s—15 min), trot—20 min and canter— 15 min, gallop—0 min

Week 14: thirty-five minutes of warm-up on the horse walker (2 m/s—5 min, 3 m/s—15 min, and 4 m/s—15 min), trot—20 min and canter— 20 min, gallop—0 min

Week 15-end: thirty-five minutes of warm-up on the horse walker (2 m/s—5 min, 3 m/s— 15 min, and 4 m/s—15 min), trot—20 min and canter— 20 min, gallop—5 min

HRmax set at 240 bpm, Trot at 50–60% HRmax, canter at 60–70% HRmax, and gallop at 70–80% HRmax.

Train for 2 days, then rest for 1 day. Trainfor 3 days, then rest for 1 day. Train in the morning and rest in the afternoon.
