# Supplementary material for: Integrating miRNA, mRNA, and Targeted Metabolomics Analyses to Explore the Regulatory Mechanism of Cardiac Remodeling in Yili Horses
Source: Biology (Basel). 2025 Nov 1;14(11):1535. doi: 10.3390/biology14111535 (PMC12650387; doi:10.3390/biology14111535)
Supplement: Supplementary file 1 [file biology-14-01535-s001.zip › Supplementary Text 3 analysis method.pdf]

Supplementary Text 3

1、PCA

Unsupervised PCA (principal component analysis) was performed by statistics function prcomp within R (www.r-project.org). The data was unit variance scaled before unsupervised PCA.

2、Hierarchical Cluster Analysis and Pearson Correlation Coefficients

The HCA (hierarchical cluster analysis) results of samples and metabolites were presented as heatmaps with dendrograms, while pearson correlation coefficients (PCC) between samples were caculated by the cor function in R and presented as only heatmaps. Both HCA and PCC were carried out by R package pheatmap. For HCA, normalized signal intensities of metabolites (unit variance scaling) are visualized as a color spectrum.

3、 Differential metabolites selected

For two-group analysis, differential metabolites were determined by VIP (VIP > 1) and P-value (P-value < 0.05, Student’s t test). VIP values were extracted from OPLS-DA result, which also contain score plots and permutation plots, was generated using R package MetaboAnalystR. The data was log transform (log2) and mean centering before OPLS-DA. In order to avoid overfitting, a permutation test (200 permutations) was performed.

4、KEGG annotation and enrichment analysis

Identified metabolites were annotated using KEGG Compound database (http://www.kegg.jp/kegg/compound/), annotated metabolites were then mapped to KEGG Pathway database (http://www.kegg.jp/kegg/pathway.html). Pathways with significantly regulated metabolites mapped to were then fed into MSEA (metabolite sets enrichment analysis), their significance was determined by hypergeometric test’s p-values.
