# Supplementary material for: Integrating miRNA, mRNA, and Targeted Metabolomics Analyses to Explore the Regulatory Mechanism of Cardiac Remodeling in Yili Horses
Source: Biology (Basel). 2025 Nov 1;14(11):1535. doi: 10.3390/biology14111535 (PMC12650387; doi:10.3390/biology14111535)
Supplement: Supplementary file 1 [file biology-14-01535-s001.zip › Supplementary Text 4.pdf]

Supplementary Text 4. Data analysis

1. Principal component analysis

Unsupervised principal component analysis (PCA) was performed using statistics function prcomp in R (www.r-project.org). The data were unit variance scaled before performing unsupervised PCA.

2. Hierarchical cluster analysis and Pearson correlation coefficients

The hierarchical cluster analysis (HCA) results of samples and metabolites were presented as heatmaps with dendrograms, while Pearson correlation coefficients (PCC) between samples were calculated using the cor function in R and visualized as heatmaps only. Both HCA and PCC were carried out using R package pheatmap. For HCA, normalized signal intensities of metabolites (after unit variance scaling) were visualized as a color spectrum.

3. Differential metabolites selected

For two-group analysis, differential metabolites were determined by variable importance in projection (VIP) value (VIP > 1) and P-value (*P*-value < 0.05, Student's t test). VIP values were extracted from OPLS-DA results, which also included score plots and permutation plots, and were generated using R package MetaboAnalystR. The data were log-transformed (log2) and mean- centered before OPLS-DA. In order to avoid overfitting, a permutation test (200 permutations) was performed.

4. KEGG annotation and enrichment analysis

Identified metabolites were annotated using KEGG Compound database (http://www.kegg.jp/kegg/compound/), and the annotated metabolites were then mapped to the KEGG Pathway database (http://www.kegg.jp/kegg/pathway.html). Pathways containing significantly regulated metabolites were subsequently subjected to metabolite sets enrichment analysis (MSEA), and their significance was determined using hypergeometric test *P*-values.
